# Transcutaneous Vagus Nerve Stimulation Does Not Affect Verbal Memory Performance in Healthy Volunteers

**DOI:** 10.3389/fpsyg.2020.00551

**Published:** 2020-04-15

**Authors:** Ann Mertens, Lien Naert, Marijke Miatton, Tasha Poppa, Evelien Carrette, Stefanie Gadeyne, Robrecht Raedt, Paul Boon, Kristl Vonck

**Affiliations:** ^1^4Brain, Department of Neurology, Institute for Neuroscience, Ghent University Hospital, Ghent, Belgium; ^2^Department of Experimental Psychology, Ghent University, Ghent, Belgium; ^3^Ghent Experimental Psychiatry (GHEP) Lab, Department of Psychiatry, Ghent University Hospital, Ghent, Belgium; ^4^Department of Psychology, University of Southern California, Los Angeles, CA, United States

**Keywords:** transcutaneous vagus nerve stimulation, verbal memory performance, word recognition memory paradigm, cognition, immediate recall, delayed recognition

## Abstract

**Introduction:**

Invasive vagus nerve stimulation (VNS) improves word recognition memory in patients with epilepsy. Recent studies with transcutaneous VNS (tVNS) have also shown positive effects on various subdomains of cognitive functioning in healthy volunteers. In this randomized, controlled, crossover study, we investigated the effect of tVNS on a word recognition memory paradigm in healthy volunteers to further investigate the potential of tVNS in the treatment of cognitive disorders.

**Methods:**

We included 41 healthy participants aged between 18 and 30 years (young age group) and 24 healthy participants aged between 45 and 80 years (older age group). Each participant completed a word recognition memory paradigm during three different conditions: true tVNS, sham, and control. During true tVNS, stimulation was delivered at the cymba conchae. Sham stimulation was delivered by stimulating the earlobe. In the control condition, no stimulation was given. In each condition, participants were asked to remember highlighted words from three test paragraphs. Accuracy scores were calculated for immediate recall after each test paragraph and for delayed recognition at the end of the paradigm. We hypothesized that highlighted words from paragraphs in the true tVNS condition would be more accurately recalled and/or recognized compared to highlighted words from paragraphs in the sham or control condition.

**Results:**

In this randomized study, tVNS did not affect the accuracy scores for immediate recall or delayed recognition in both age groups. The younger group showed significantly higher accuracy scores than the older group. The accuracy scores improved over time, and the most recently learned words were better recognized. Participants rated true tVNS as significantly more painful; however, pain was not found to affect accuracy scores.

**Conclusion:**

In this study, tVNS did not affect verbal memory performance in healthy volunteers. Our results could not replicate the positive effects of invasive VNS on word recognition memory in epilepsy patients. Future research with the aim of improving cognitive function should focus on the rational identification of optimized and individualized stimulation settings primarily in patients with cognitive deficits.

## Introduction

There is an ever-increasing scientific interest in the vagus nerve as a potential target for memory modulation. Age-related declines are seen in short-term memory functioning and in free recall (retrieval) probably due to general slowing, reduced processing resources, loss of inhibitory functions, and lack of cognitive control (Luo and Craik, 2008). However, the brain is able to reorganize itself during aging, learning, and following damage, a process defined as neural plasticity. This concept has stimulated the development of new treatment options for cognitive decline, aiming to enhance this plastic potential (Duffau, 2006). The formation of declarative or explicit memory requires three essential processes: learning-encoding, consolidation-storage, and retrieval (Tulving, 1983). After information is perceived, it enters the memory system through the short-term memory function (Baddeley and Hitch, 1974) in which a small amount of information can be held active, as long as attention to the stimulus is maintained. Subsequently, information is stored in the long-term memory system, depending on the depth and elaboration of processing of the information. Retrieval of the stored information refers to the activation of the correct information from the long-term memory into the short-term memory, while suppressing the incorrect information (Shiffrin and Steyvers, 1997). It has been well documented that arousal shortly following a learning experience, during the process of memory consolidation, can modulate the storage of information (Cahill and McGaugh, 1996; McGaugh, 1966; McGaugh, 2015). Although this process has not been fully elucidated, preclinical research suggests that the vagus nerve plays a crucial role in transmitting the signals of peripheral neuromodulators associated with arousal to brain structures involved in memory storage (Williams and McGaugh, 1993; Nogueira et al., 1994; Hassert et al., 2004).

In 1999, Clark et al. (1999) demonstrated that stimulation of the vagus nerve, by means of an implanted device to treat drug-resistant epilepsy patients, was able to significantly enhance verbal memory performance when stimulation was delivered during the consolidation phase of a memory task. In 2006, Ghacibeh et al. (2006) found that VNS improved the retention of information by enhancing consolidation rather than by affecting memory retrieval, concluding that VNS specifically interacts with the processes underlying memory consolidation.

The vagus nerve projects to the nucleus of the solitary tract and consequently activates the noradrenergic neurons in the locus coeruleus and cholinergic neurons in the nucleus basalis, resulting in the release of norepinephrine (NE) and acetylcholine in wide areas of the cortex (Gu, 2002; Hassert et al., 2004; Roosevelt et al., 2006; Follesa et al., 2007; Nichols et al., 2011; Raedt et al., 2011). NE subsequently causes a release of serotonin by activating alpha-1-adrenergic receptors in the dorsal raphe nucleus (Manta et al., 2009). These neurotransmitters are known to facilitate neural plasticity, a key mechanism in many behavioral and cognitive processes (Gu, 2002; Duffau, 2006). Other neurotransmitters presumably involved in the mechanism of action of VNS are gamma-aminobutyric acid (GABA) and aspartate (Hammond et al., 1992; Ben-Menachem et al., 1995). Long-term potentiation is considered the major cellular mechanism of memory formation. As NE is known to facilitate this early long-term potentiation through the activation of beta-noradrenergic receptors, the VNS-induced NE release has been proposed as a possible mechanism of modulating memory performance (Harley, 2007; Mueller et al., 2008). These findings have given rise to an increasing interest in neuromodulation as a potential treatment for cognitive disorders. Currently available treatment options for cognitive dysfunction, including pharmacotherapy and psychosocial interventions, have shown limited effects on cognition (Perng et al., 2018). Based on the potential of VNS to modulate memory formation and the positive effects seen in epilepsy patients, VNS has been investigated as a potential treatment option for conditions associated with cognitive decline with promising results (Sjogren et al., 2002; Merrill et al., 2006).

Recently, noninvasive treatment options have gained interest, aiming to achieve the same effects as invasive VNS without the need for an invasive procedure. Transcutaneous vagus nerve stimulation (tVNS) represents a noninvasive neurostimulation modality that targets the receptive field of the auricular branch of the vagus nerve, located at the outer part of the ear (Ellrich, 2019). Functional imaging studies have shown that tVNS leads to activation of intracranial structures similar to the ones activated by invasive VNS, suggesting potential for evoking similar effects in a less invasive manner (Yakunina et al., 2017). Seeking to replicate the cognitive effects of invasive VNS seen in patients, several clinical studies have investigated the modulatory effect of tVNS on cognitive functioning in healthy volunteers (Jacobs et al., 2015; Sellaro et al., 2015, 2018; Steenbergen et al., 2015; Beste et al., 2016; Colzato et al., 2017, 2018a,b; Jongkees et al., 2018). Recent studies demonstrated that tVNS affected post-error slowing (Sellaro et al., 2015), response selection functions (Steenbergen et al., 2015; Jongkees et al., 2018), response speed when two actions were executed in succession (Steenbergen et al., 2015), divergent thinking (Colzato et al., 2018a), and emotion recognition (Colzato et al., 2017; Sellaro et al., 2018). tVNS also significantly influenced inhibitory control processes (Beste et al., 2016) and decreased flow experience during a task (Colzato et al., 2018b). A study by Jacobs et al. (2015) was the first study to investigate the effect of tVNS on memory performance. They demonstrated that a single session of tVNS enhanced associative memory performance in older healthy volunteers, measured by means of an associative face–name memory task.

In this study, we aimed to replicate the positive effect of invasive VNS on verbal memory performance seen in epilepsy patients. Therefore, we investigated whether tVNS is able to improve verbal memory in younger as well as older healthy participants by applying stimulation during the consolidation phase of a word recognition memory paradigm. We hypothesized that tVNS, as compared to sham stimulation and control, would enhance verbal memory performance.

## Materials and Methods

### Participants

The effect of tVNS on memory function was investigated in healthy volunteers belonging to two different age groups: young individuals between 18 and 30 years and older individuals between 45 and 80 years. Forty-one participants were included in the young age group and 24 participants in the older age group. Participants were recruited through flyers and an online recruitment system. Subjects were excluded in case of a history of cardiac disease, substance abuse or dependence, treatment with psychoactive drugs, a history of neurological or psychiatric disorders, pregnancy, and presence of an active implanted device (e.g., pacemaker, VNS, cochlear implant) or cerebral shunt. In the older age group, cognitive status was examined with the Montreal Cognitive Assessment (MoCA) test battery. Participants with a MoCA score lower than 24 were also excluded. Written informed consent was obtained from each participant before the beginning of the experimental session. Participants were instructed to have a light breakfast or lunch on the day of the experimental session and to avoid caffeine 2 h before. During the experimental session, the participant was not allowed to eat. Drinks were limited to water. Before conducting the experimental session, each participant was asked to fill out a demographic questionnaire. At the end of the experimental session, subjects received a gift certificate with a value of 20 euro for participating.

The study protocol was reviewed and approved by the ethics committee of Ghent University Hospital and conformed to the ethical standards of the Declaration of Helsinki.

### Procedure

**FIGURE 1:**
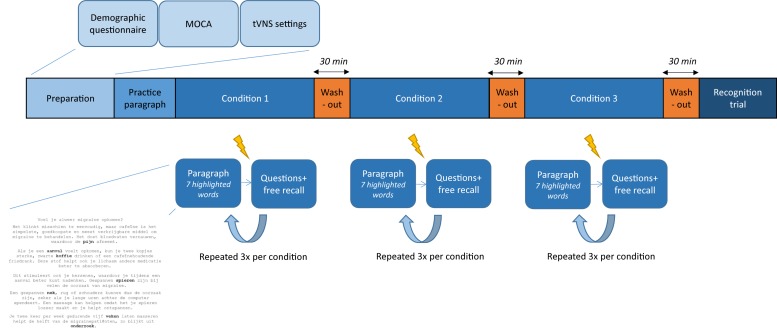
Overview of the study. After preparation of the experimental session and one practice paragraph, the word recognition memory paradigm is conducted. Three experimental conditions are used, separated by a washout period of 30 min. Stimulation (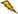) is delivered 2 min after reading a paragraph and followed by two questions and a free recall task. This is repeated three times per condition. At the end of the paradigm, participants perform a recognition test.

This (sham-)controlled, randomized, crossover, within-subjects study investigated the effect of tVNS on verbal memory performance in healthy volunteers. An overview of the study protocol is presented in Figure 1. First, the investigator and participant went through the inclusion criteria and informed consent form. Participants were included in the study after signing the informed consent form. Second, demographic data were collected through a questionnaire. For the participants in the older age group, cognitive functioning was also evaluated by administering the MoCA. After the appropriate tVNS amplitude was chosen (according to the threshold method or set to 0.5 mA), each subject conducted the word recognition memory paradigm in three different conditions: true stimulation, sham stimulation (active control), and no stimulation (control). At the end of the paradigm, a word recognition task was performed. A washout period of 30 min was implemented between experimental conditions and before the recognition task. During these breaks, participants were asked to perform a relaxing activity. The order of experimental conditions was randomized across subjects. All the experimental sessions were conducted at the Neurology Department at Ghent University Hospital in a neutral examination room. The experimental session lasted 3 h including breaks and could take place in the morning or in the afternoon, depending on the availability of the participant.

### Transcutaneous Vagus Nerve Stimulation

Stimulation was delivered by means of the NEMOS^®^ tVNS device (Cerbomed, Erlangen, Germany) which targets the cutaneous receptive field of the auricular branch of the vagus nerve at the outer ear. This external device consists of an auricular electrode connected to a control unit. The electrode is attached to an earplug to ensure that the electrode is placed on the cymba conchae. Sham stimulation was delivered by inverting the earplug and placing the electrode on the earlobe. Stimulation at this location will cause the same tingling sensation but will not activate the vagus nerve (Peuker and Filler, 2002; Kraus et al., 2013).

Based on previously published tVNS protocols (Steenbergen et al., 2015; Colzato et al., 2018a, b), stimulation intensity was set to 0.5 mA in 16 participants. As perceived and tolerated stimulation intensity varies across participants, we decided to set the stimulation intensity to the maximum tolerated output in the other 49 participants by using the threshold method. Before the beginning of the memory task, stimulation was increased in steps of 0.10 mA until the participant felt a tingling sensation. Stimulation was then further increased until the participant reported pain and finally decreased 0.10 mA below the pain threshold. This stimulation output was noted and used throughout the experimental session for both the sham and true tVNS conditions. Stimulation was delivered for 30 s during the consolidation phase of the memory task. Frequency was set to 25 Hz and pulse width to 250 μs.

The participants were informed that stimulation would be given during the experimental session by means of the tVNS device. Possible side effects of stimulation were discussed (e.g., pain, redness of the skin, itching) (Mertens et al., 2018). The participants were not informed about the type of stimulation (sham versus true) and expected outcome.

### Word Recognition Memory Paradigm

The memory task in this study (Figure 1) was based on the word recognition memory paradigm in the study by Clark et al. (1999). It was designed with E-prime software (Psychology Software Tools Inc., Pittsburgh, PA, United States) and conducted on a laptop with a 14-inch screen (Dell, Windows 7). A short introduction was given by the investigator before starting the memory task. Written instructions were displayed on the laptop during the experimental session. Participants were instructed to silently read fragments of text paragraphs displayed on the screen. A practice paragraph was given before the beginning of the memory task to familiarize the participant with the testing procedures. The paragraphs were chosen from the “wablieft krant,” an online journal known for its low difficulty level. One paragraph was divided into five to six fragments, displayed separately on the computer screen. Participants could continue to the next fragment by pressing the space bar. In each paragraph, seven words were highlighted. Participants were asked to read the paragraph thoroughly and memorize the highlighted words. Two minutes after finishing reading a paragraph, stimulation was delivered during 30 s. No stimulation was delivered in the control condition. In accordance to the study by Clark et al. (1999), stimulation was delivered after 2 min in order to stimulate during the consolidation phase of memory formation. Immediately after stimulation, participants were asked to answer two questions on the content of the paragraph and to write down as many of the highlighted words as possible (immediate free recall). Afterwards, participants were asked to rate pain during the stimulation. In the first 25 participants, a Likert scale from 1 to 9 was used. However, as participants reported difficulties in using this scale, the Wong–Baker FACES Pain Rating Scale ranging from 0 to 10 was used for the remaining 40 participants. The pain ratings of the first 25 participants were converted to fit the new scale. Three consecutive paragraphs with associated questions were merged into one text file. For each experimental condition, one text file was used, leading to a total of 21 highlighted words to be remembered and six questions to be answered per condition. Each subject conducted the paradigm sequentially in three conditions: true tVNS, sham, and control (no stimulation), summing up to nine paragraphs, 63 highlighted words, and 18 questions throughout the experimental session. Both the order of text files and conditions were randomized across subjects. A 30-min break was added after each condition to ensure washout between the different conditions. After completing all three conditions and adding a last break of 30 min, the recognition task was performed. During this final task, all 63 highlighted words as well as 63 related words and 63 non-related words were displayed on the computer screen in a randomized order. Each participant was asked to recognize target words and distinguish them from non-target words by pressing a green button when a target word was displayed and pressing a red button for a non-target word.

### Outcome Measures

Primary outcome measures were accuracy scores on the immediate recall tests (after each test paragraph) and accuracy scores on the delayed recognition test (at the end of the paradigm). Regarding delayed recognition accuracy scores, only correct categorization of the highlighted words (hits) was compared. The categorization of related and unrelated novel words was not considered. We hypothesized that highlighted words from paragraphs in the true tVNS condition would be more accurately recalled and/or recognized compared to highlighted words from paragraphs in the sham or control condition.

### Data Analysis

For each participant, immediate recall scores were calculated by the investigator based on the number of correct words the participant noted after each paragraph. This resulted in a mean accuracy score (percentage) for each stimulation condition. Delayed recognition scores were calculated using R statistical software (R Core Team, 2017), resulting in accuracy scores for correct categorization of the highlighted words (hit or miss) on a single trial level.

The data analysis was conducted using R with lme4 (Bates et al., 2015) to perform generalized linear mixed effects (GLMEs) analyses. In case the dependent variable was dichotomous (categorization accuracy for delayed recognition test), we used logistic regression analyses. Both for the fixed and random effects, the chi-square statistics and the corresponding *p*-values were acquired by the likelihood ratio test. The dependent variable was the accuracy on the immediate recall and delayed recognition test. The independent variable was the stimulation condition (tVNS, sham, and control). The study order (block 1, block 2, block 3), the text file (file1, file2, file3), the stimulation intensity, pain score, gender, age, and years of education were also taken into account, as well as the MoCA score for the older group. As our dataset is relatively small, R failed to converge when making a full model with all the fixed and random effects. Therefore, we chose to start from the null model with a random intercept for subject and compare it with the model with the effect at test. This was done for all variables of interest for both the fixed effects and the random effects. Hereby, we can test if a variable is an important predictor in its own right, independent of the presence of any other variables. A significance level of *p* < 0.05 was adopted for all statistical tests.

Data analysis was conducted for both age groups separately as well as for all participants together.

## Results

### Demographics

Forty-one participants (20 males) were included in the young age group with a mean age of 22.20 years (+1.97) and mean years of education of 15.44 years (+2.12). In the older age group, 24 participants were included, of whom seven were males. The mean age was 55.13 years (+6.59), and the mean years of education were 15.21 years (+2.05). The younger and the older age groups did not significantly differ from each other on gender [χ^2^ (1, *N* = 65) = 1.66, *p* = 0.20] and years of education [Welch’s *t*(49.21) = 0.43, *p* = 0.67].

### Stimulation and Pain Report

The mean stimulation intensity was 0.54 mA (±0.21) in the younger group and 0.57 mA (±0.12) in the older group. Both groups did not significantly differ in stimulation intensity [Welch’s *t*(62.99) = 0.35, *p* = 0.72]. Stimulation intensity had a significant effect on reported pain level [χ^2^ (1, *N* = 65) = 7.82, *p* = 0.0051], with significantly higher pain scores after lower stimulation. There was a trend for higher reported pain levels in the younger group (1.08 ± 1.71) compared to the older group (0.57 ± 1.10), but this difference was not significant [χ^2^ (1, *N* = 65) = 3.70, *p* = 0.055]. A significant effect of experimental condition on pain reports [χ^2^ (1, *N* = 65) = 10.31, *p* = 0.0013] was found, showing that true tVNS led to a significantly higher pain score than sham [Welch’s *t*(123.22) = 2.68, *p* = 0.0083], and sham led to a significantly higher pain score than the control condition [Welch’s *t*(113.13) = 2.83, *p* = 0.0055].

### Immediate Recall

We hypothesized to find an effect of experimental condition on immediate recall scores, more specifically, higher immediate recall accuracy scores for the true tVNS condition compared to the sham and control conditions. We first analyzed the data of the younger and older groups separately and then compared both groups.

#### Young Age Group

**FIGURE 2:**
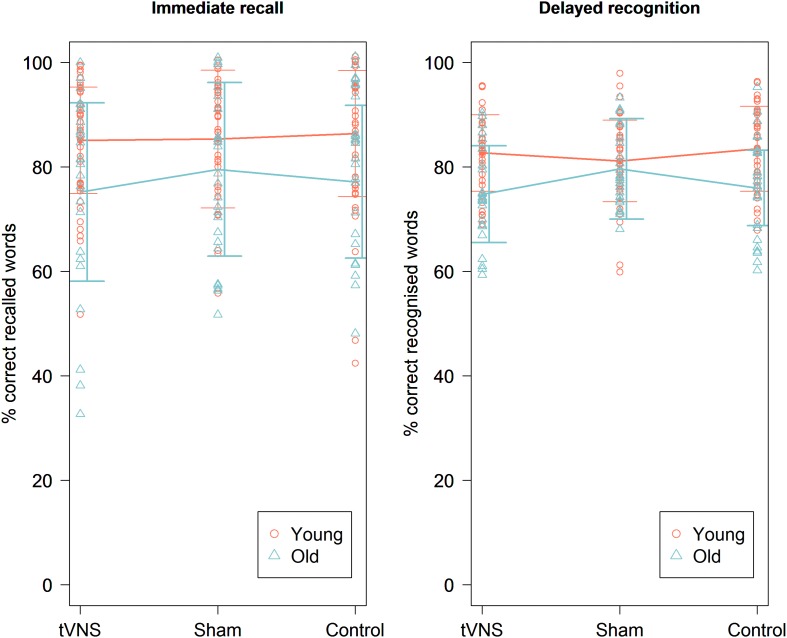
Immediate recall **(left)** and delayed recognition **(right)** accuracy scores in percentage for the three experimental conditions for the young and old age group. There was no significant effect of experimental condition on immediate recall and delayed recognition scores in both age groups. Line plots represent mean scores. Error bars represent standard error.

**FIGURE 3:**
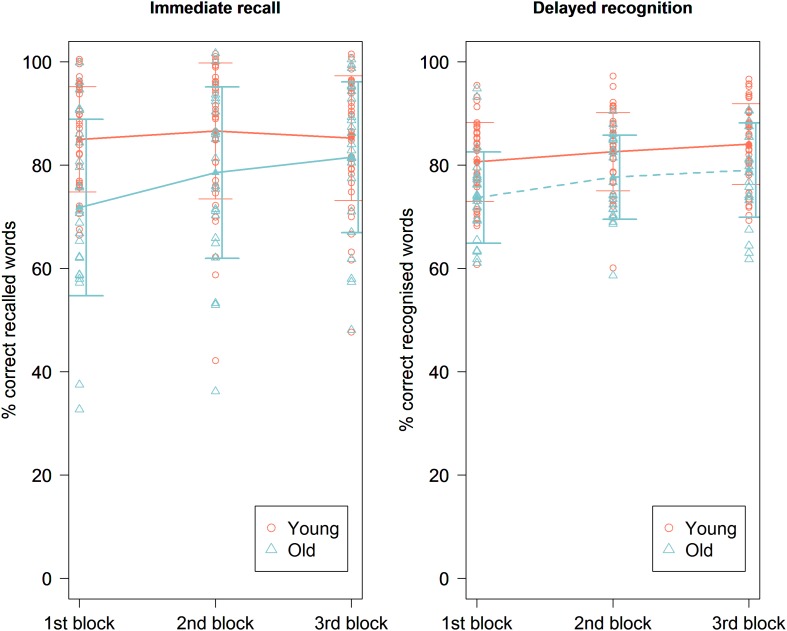
Immediate recall **(left)** and delayed recognition **(right)** accuracy scores in percentage during the three blocks of the experimental session for the young and the old group. A significant effect of order was seen on immediate recall scores in the old age group and on delayed recognition scores in both age groups, showing significantly higher scores toward the end of the paradigm. Line plots represent mean scores. Error bars represent standard error.

**FIGURE 4:**
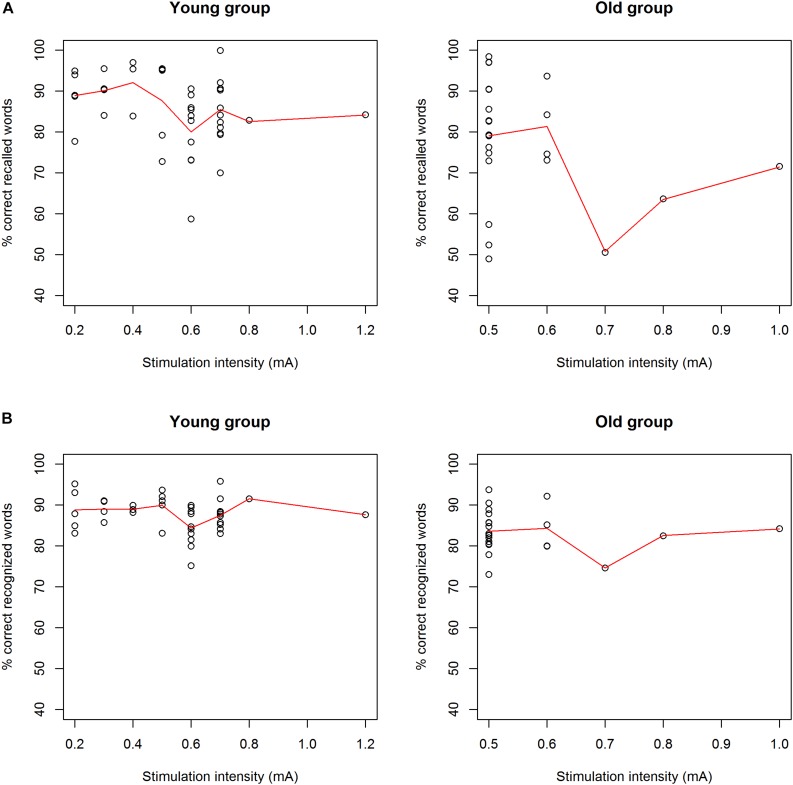
**(A)** Correlation between immediate recall scores and stimulation intensity for the young group (left) and the old group (right). There was no correlation between stimulation intensity and immediate recall scores in both age groups. **(B)** Correlation between delayed recognition scores and stimulation intensity for the young group (left) and the old group (right). There was no correlation between stimulation intensity and delayed recognition scores in both age groups.

The mean accuracy score on the immediate recall test was 85.64% (±11.81%). We found no main effect of experimental condition [χ^2^ (1, *N* = 41) = 0.37, *p* = 0.83] (Figure 2). There was also no main effect of order [χ^2^ (1, *N* = 41) = 0.011, *p* = 0.92] (Figure 3). There was a significant main effect for the specific text they had to memorize, with highest accuracy scores for text file 2 [χ^2^ (1, *N* = 41) = 14.27, *p* = 0.0008]. There were no significant random effects. There was no effect of pain report, stimulation intensity (Figure 4), age, gender, or education level on accuracy.

#### Old Age Group

The mean accuracy score on the immediate recall test was 77.31% (±16.43%). We found no main effect of experimental condition [χ^2^ (1, *N* = 24) = 2.56, *p* = 0.11] (Figure 2). There was a significant main effect of order [χ^2^ (1, *N* = 24) = 14.76, *p* = 0.00012], showing that the accuracy improved over time (Figure 3). Similar to the younger group, a significant main effect was found for the specific text they had to memorize, with highest accuracy scores for text file 2 [χ^2^ (1, *N* = 24) = 21.07, *p* < 0.001]. There was an effect of age on accuracy [χ^2^ (1, *N* = 24) = 24.35, *p* < 0.0001], showing higher accuracy scores for younger participants. There was also a significant effect of MoCA score on accuracy [χ^2^ (1, *N* = 24) = 4.88, *p* = 0.027], showing that participants with a higher MoCA score obtained higher recall scores. There were no significant random effects. There was no effect of pain reports, stimulation intensity (Figure 4), gender, or years of education on the immediate recall score.

#### Both Age Groups

The mean accuracy score on the immediate recall test when combining both datasets was 82.56% (±14.24%). Age group (young versus old) had a significant main effect on recall accuracy, with significantly higher scores in the young age group [χ^2^ (1, *N* = 65) = 8.55, *p* = 0.0034]. Regarding the effects of experimental condition on accuracy, we found no main effect of condition [χ^2^ (1, *N* = 65) = 1.16, *p* = 0.28]. A significant main effect of order was found [χ^2^ (1, *N* = 65) = 4.66, *p* = 0.031], showing that the accuracy improved over time. There was also a significant main effect for the specific text they had to memorize, with highest accuracy scores for text file 2 [χ^2^ (1, *N* = 65) = 32.62, *p* < 0.0001]. There were no significant random effects. There was no effect of pain report, stimulation intensity, gender, or education level on accuracy.

### Delayed Recognition

During the recognition task, delayed recognition accuracy scores were obtained for correct categorization of the highlighted words. The categorization of related and unrelated novel words was not considered. We hypothesized that higher accuracy scores on the delayed recognition task would be associated with the true tVNS condition as compared to the sham and control conditions. We first analyzed the data of the younger and older groups separately and then compared both groups.

#### Young Age Group

The mean accuracy score on the delayed recognition test was 73.17% (±11.26%). No significant main effect of experimental condition was found [χ^2^ (1, *N* = 41) = 0.01, *p* = 0.90] (Figure 2). There was a significant main effect of order [χ^2^ (1, *N* = 41) = 12.72, *p* = 0.00036], showing a recency effect where the most recent learned words are better recognized (Figure 3). There was also a significant random effect of order, showing that the strength of this recency effect differed across participants [χ^2^ (1, *N* = 41) = 7.18, *p* = 0.028]. There was no significant effect of text. We also found no effect of stimulation intensity (Figure 4), pain report, age, gender, or educational level on accuracy scores.

#### Old Age Group

The mean accuracy score on the delayed recognition test was 65.67% (±13.29%). No significant main effect of experimental condition was found (χ^2^ (1, *N* = 24) = 0.99, *p* = 0.32) (Figure 2). There was a significant main effect of order [χ^2^ (1, *N* = 24) = 25.79, *p* < 0.0001], showing again a recency effect (Figure 3) as well as a significant random effect of order [χ^2^ (1, *N* = 24) = 13.75, *p* = 0.0010]. No significant effect of text was found. A trend was seen toward an effect of age on accuracy scores showing that the younger participants are, the higher their score is [χ^2^ (1, *N* = 24) = 2.86, *p* = 0.091]. There was no effect of stimulation intensity (Figure 4), pain report, gender, educational level, or MoCA score on accuracy scores.

#### Both Age Groups

The mean accuracy score on the delayed recognition test was 70.40% (±12.49%). No significant main effect of experimental condition was found [χ^2^ (1, *N* = 65) = 0.18, *p* = 0.67]. Also for both groups combined, a significant main effect of order [χ^2^ (1, *N* = 65) = 34.12, *p* < 0.0001] as well as a significant random effect of order [χ^2^ (1, *N* = 65) = 16.86, *p* = 0.00022] was found, showing a recency effect that differed across participants. There was a significant effect of text on accuracy scores [χ^2^ (1, *N* = 65) = 7.41, *p* = 0.025] with significantly lower scores on text file 1. A significant effect of group [χ^2^ (1, *N* = 65) = 13.21, *p* = 0.00028] was seen, showing that the younger group scored significantly higher on the delayed recognition test than the older group. No effect of stimulation intensity, pain report, gender, or educational level on accuracy scores was seen.

### Power Analysis

A *post hoc* power analysis was conducted as the effect size could not be established before the beginning of the clinical study. The effect size (tVNS compared to control) was *d* = 0.19 for the immediate recall and *d* = 0.03 for the delayed recognition. By means of the package WebPower (Zhang and Yuan, 2018) in R, we used a function specifically for regression models to determine the sample size with a power of 0.8 and alpha set to 0.05. Regarding the immediate recall, a sample size of 43 participants was required to obtain a power of 80%. For the delayed recognition, a sample size of 263 participants was required to obtain a power of 80%.

## Discussion

While VNS was previously shown to improve performance on memory paradigms, we did not find a significant effect of tVNS on verbal memory performance in young and older healthy participants. Differences in the study methodology may underlie the different outcomes with regard to the effects of VNS on memory function (see also Table 1).

**TABLE 1:** Overview of study characteristics of this clinical study and previous research investigating the effect of VNS on memory performance.

	This study	Clark et al. (1999)	Jacobs et al. (2015)
Study design	Within-subjects	Within-subjects	Within-subjects
Study population	Healthy volunteers	Epilepsy patients	Healthy older volunteers
	young age group
	old age group
Sample size	65	10	30
	41 young age group
	24 old age group
Memory function	Immediate recall	Immediate recall Delayed recognition	Name–face association
	Delayed recognition	Delayed recognition
Device	tVNS (NEMOS)	Invasive VNS	tVNS (TENStem)
Stimulation parameters			
Output current	According to threshold	0.5–1.5 mA	5 mA
Frequency	25 Hz	30 Hz	8 Hz
Pulse width	250 μs	500 μs	200 μs
Duration	30 s	30 s	17 min
Washout period	30 min	unknown	>7 days
Number of conditions	3 (tVNS, sham, control)	2 (VNS, sham)	2 (tVNS, sham)

We investigated healthy participants, while in the study by Clark et al. (1999), the effect of VNS on memory performance was evaluated in epilepsy patients. It has been shown that epilepsy is associated with cognitive comorbidities including memory impairment (Butler and Zeman, 2008; Helmstaedter and Witt, 2017). A lower baseline performance in epilepsy patients may be more prone to improvement as compared to healthy volunteers, in whom the verbal memory performance test cannot be further improved, a feature described as “the ceiling effect.” The study by Jacobs et al. (2015) also included only healthy volunteers, but inclusion was restricted to older individuals with a higher mean age compared to our participants in the old age group (60.57 years ± 2.54 versus 55.13 years ± 6.59), with potentially lower baseline memory scores again more susceptible to improvement.

In our study, a noninvasive device for targeting the vagus nerve was used. However, more effective stimulation of the vagal afferent pathway may be achieved when the vagus nerve is targeted directly by means of an implanted device. The optimal stimulation location and parameters of tVNS have not been elucidated. We chose to target the cymba conchae as this region is exclusively innervated by the auricular branch of the vagus nerve (Peuker and Filler, 2002), and stimulation at this location produced a significant activation of intracranial structures similarly affected by invasive VNS (Yakunina et al., 2017).

Previous research has shown that moderate levels of stimulation were most efficient for improving memory performance, whereas low and high levels of stimulation caused no improvement or even deterioration, visualized by an inverted U-curve (Clark et al., 1999). In this study, we did not find a significant correlation between stimulation intensity and accuracy scores on immediate recall and delayed recognition (Figure 4). Participants who could tolerate higher output currents did not perform better than participants receiving a lower stimulation intensity. The previously described inverted U-curve could also not be confirmed by our results. However, we do emphasize that we did not investigate the effect of different stimulation intensities within subjects as was conducted in the study by Clark et al. (1999). The optimal stimulation intensity of tVNS for improving memory performance remains to be elucidated. Therefore, it is possible that subjects in this study were not stimulated at individually optimized levels of intensity.

Stimulation was delivered for only 30 s during the consolidation phase of a memory task, analogous to the invasive VNS protocol in Clark et al. (1999). However, 30 s of tVNS may be insufficient for a noninvasive device to effectively stimulate the vagal afferent pathway. As long-term potentiation is considered the most important mechanism of memory formation, longer and more repetitive stimulation of the vagus nerve might be required to effectively modulate hippocampal processes. In addition, some participants only tolerated very low output currents which could have been too low to sufficiently activate vagal afferent fibers. In the study by Jacobs et al. (2015), a significant effect on associative memory was found with a different tVNS device that continuously stimulated the inner side of the tragus during 17 min. The longer stimulation duration used in the Jacobs et al. (2015) study may prove more effective at modulating memory performance. However, it could also be possible that tVNS only interacts with specific memory functions, such as associative memory, and is not able to improve immediate recall or delayed recognition. In 2016, Burger et al. investigated the effect of tVNS on fear extinction, a process that is also highly dependent on memory formation (Burger et al., 2016, 2017, 2018; Verkuil et al., 2017). A significant acceleration of fear extinction learning was seen after tVNS; however, this did not lead to better retention of extinction memory. By further investigating the mechanism of action of (t)VNS, potential targets of memory function and optimal conditions for intervention could be identified.

In this study, we used a relatively short interval between the conditions. Thirty minutes may have been too short to ensure complete washout. Due to the setup of the word recognition memory paradigm, all conditions had to be conducted in one experimental session on the same day. To date, studies investigating the enduring effects of invasive VNS on NE show inconsistent results; some authors describe completely transient effects (Roosevelt et al., 2006), while others demonstrated elevated NE levels up to 2 h after stimulation (Hassert et al., 2004). To our knowledge, the enduring effect of tVNS has not been studied.

In contrast to previous research, we compared true tVNS to both a sham stimulation and control condition. The use of sham stimulation by means of stimulating the earlobe is under discussion (Keute et al., 2018; Rangon, 2018). Not including a sham stimulation would lead to blinding issues as participants can clearly distinguish the true condition from the control. A sham stimulation is also necessary to ensure that effects are caused by activating the vagal trajectory and not merely by the sensation of electrical current through the trigeminal nerve (Keute et al., 2018). As true tVNS did not significantly alter verbal memory performance compared to both a sham and control condition, we concluded that these results were not confounded by insufficient blinding or by sham-induced activation. Participants did report higher pain scores during true tVNS than sham, which could possibly impact their performance. However, we did not find a significant effect of pain on accuracy scores in both age groups.

A limitation of this study is the sample size. A *post hoc* power analysis indicated that the sample size of this study was sufficient to reliably investigate the effect of tVNS on immediate recall but should be extended to 263 participants for delayed recognition. This should be considered when interpreting the results for delayed recognition.

Although no effect of stimulation was found in this study, several experimental and demographic factors were identified that significantly affected verbal memory performance.

In the older age group, a practice effect was found with a significant increase in immediate recall accuracy scores throughout the paradigm. This practice effect was not seen in the younger age group. In all volunteers, highlighted words that were presented at the last condition of the paradigm were more easily recognized than highlighted words at the beginning, demonstrating a recency effect. A significant effect of text file was also found in both age groups, indicating that the highlighted words in some paragraphs could be more easily remembered than others. This effect was unexpected as all paragraphs were chosen from the same online journal involving health-related topics, and the highlighted words were controlled for frequency and concreteness ratings (Fliessbach et al., 2006; Keuleers et al., 2010; Lohnas and Kahana, 2013; Brysbaert et al., 2014). As we counterbalanced the order of intervention (active tVNS was randomly delivered as first, second, or third intervention) and text files across participants, these practice, recency, and text effects should not have interfered with our results. These findings emphasize the difficulties in designing a reliable neuropsychological study and the importance of counterbalancing conditions and test versions across participants.

Gender and years of education did not have an effect on memory performance. In the older age group, a higher MoCA score improved accuracy scores on immediate recall. Delayed recognition scores also seemed to increase with higher MoCA score, but this effect was not significant. Only in the older age group was a significant correlation between age and accuracy score on immediate recall found, with lower test scores as age increased. When comparing both age groups, we found significantly higher accuracy scores on immediate recall as well as delayed recognition in the young age group compared to the older age group. These findings demonstrate that aging above 45 years significantly reduces verbal memory performance.

This study does not find evidence that noninvasive targeting of the vagus nerve improves verbal memory performance in young and older healthy volunteers. Methodological issues potentially underlying the absence of effects have been discussed. Further research to investigate the potential of targeting vagus nerve fibers noninvasively to improve cognitive function is required. As optimal stimulation parameters have not been elucidated, future research should focus on the effect of different stimulation settings in an individualized way in order to define the most efficient stimulation parameters.

## Data Availability Statement

The datasets generated for this study are available on request to the corresponding author.

## Ethics Statement

The studies involving human participants were reviewed and approved by the Ethics committee of Ghent University Hospital, Ghent, Belgium. The patients/participants provided their written informed consent to participate in this study. Written informed consent was obtained from the individual(s) for the publication of any potentially identifiable images or data included in this article.

## Author Contributions

AM was responsible for data acquisition and drafting of the manuscript. LN conducted the data processing and statistical analysis. MM, TP, EC, SG, RR, PB, and KV proofread the manuscript. The experimental sessions were conducted under the supervision of MM and KV.

## Conflict of Interest

EC has received travel and registration grants from Elekta Neuromag Oy to participate in conferences and workshops on MEG. PB has received consultancy and speaker fees from UCB Pharma, LivaNova, Medtronic, and Eisai. KV and PB have received speaker fees from Livanova. PB, KV, and RR have received free devices for research studies in normal volunteers and preclinical studies from LivaNova, Cerbomed, Neurosigma, Medtronic. The remaining authors declare that the research was conducted in the absence of any commercial or financial relationships that could be construed as a potential conflict of interest.
